# The Effect of Natural Substances Contained in Bee Products on Prostate Cancer in In Vitro Studies

**DOI:** 10.3390/molecules28155719

**Published:** 2023-07-28

**Authors:** Przemysław Woźniak, Anna Kleczka, Krzysztof Jasik, Agata Kabała-Dzik, Radosław Dzik, Jerzy Stojko

**Affiliations:** 1 Department of Toxicology and Bioanalysis, Faculty of Pharmaceutical Sciences in Sosnowiec, Medical University of Silesia in Katowice, Ostrogórska 30, 41-200 Sosnowiec, Poland; przemekw1971@gmail.com (P.W.); jstojko@sum.edu.pl (J.S.); 2 Department of Pathology, Faculty of Pharmaceutical Sciences in Sosnowiec, Medical University of Silesia in Katowice, Ostrogórska 30, 41-200 Sosnowiec, Poland; akleczka@sum.edu.pl (A.K.); kjasik@sum.edu.pl (K.J.); 3 Faculty of Biomedical Engineering, Department of Biosensors and Processing of Biomedical Signals, Silesian University of Technology, Roosevelta 40, 41-800 Zabrze, Poland; radoslaw.dzik@gmail.com

**Keywords:** prostate cancer, flavonoids, apoptosis

## Abstract

Prostate cancer is a common cancer in men in older age groups. The WHO forecasts an increase in the incidence of prostate cancer in the coming years. Patients may not respond to treatment, and may not tolerate the side effects of chemotherapy. Compounds of natural origin have long been used in the prevention and treatment of cancer. Flavonoids obtained from natural products, e.g., propolis, are compounds with proven antibacterial and antiviral efficacy which modulate the immune response and may be useful as adjuvants in chemotherapy. The main aim of the present study was to evaluate the cytotoxic and pro-apoptotic properties of selected flavonoids on prostate cancer cells of the LNCaP line. The compounds used in this study were CAPE, curcumin (CUR), and quercetin (QUE). Mitochondrial and lysosome metabolism was assessed by the XTT-NR-SRB triple assay as well as by the fluorescent staining techniques. Staining for reactive oxygen species was performed as well. The experiment showed that each of the tested compounds has a cytotoxic effect on the LNCaP cell line. Different types of cell death were induced by the tested compounds. Apoptosis was induced by quercetin, while autophagy-specific changes were observed after using CAPE. Compounds obtained from other bee products have antiproliferative and cytotoxic activity against LNCaP prostate cancer cells.

## 1. Introduction

Worldwide, prostate cancer is the most commonly diagnosed malignancy and the fifth leading cause of cancer-related deaths in men. The most common histopathological subtype of primary prostate cancer is lobular adenocarcinoma, diagnosed in over 90% of patients. Other histological types of cancer—squamous cell carcinoma, urtoelial carcinoma, and neuroendocrine tumors—occur very rarely, and involve metastatic changes [1,2]. The incidence of prostate cancer has increased in recent decades, mainly due to increased detection and more widespread use of prostate-specific antigen (PSA) tests. Prostate cancer mortality rates have remained relatively constant over this period [3]. More than 70% of prostate cancer patients come from developed countries [4].

It is estimated that the incidence of prostate cancer will increase worldwide by 2040. The highest incidence of prostate cancer is likely to be recorded in Africa (+120.6%), Latin America and the Caribbean (+101.1%), and Asia (100.9%). The lowest incidence is projected for Europe (+30.1%). The increase in morbidity rates is mainly related to increased life expectancy. It is predicted that increased incidence of prostate cancer may be related to a “westernized” lifestyle. Increasing obesity, lack of physical activity and changing dietary habits may increase the risk of cancer in African and Asian countries [5,6].

Prostate cancer can be asymptomatic in its early stages and is often benign, requiring minimal treatment or simply observation. The most common complaints reported by patients are difficulty urinating, increased frequency of micturition, and nocturia. However, all these symptoms can result from benign prostatic hyperplasia. More advanced stages of the disease are most often manifested by urinary retention and back pain, as the bones are the most common location of prostate cancer metastases [3,7].

Diagnosis is primarily based on prostate-specific antigen (PSA) and transrectal ultrasound-guided prostate biopsies (TRUS). Elevated serum PSA levels occur in about 20% of patients without prostate cancer; it is worth noting, however, that about 30% of cancer patients may not have elevated PSA [8,9]. Histopathological examination determines the histopathological type of cancer and the degree of malignancy of the cancer as measured on the Gleason scale. This scale includes grading in the range of 2 to 10, with 2 corresponding to the least malignant tumors and 10 to the highest. The Gleason score is essential in determining the prognosis of prostate cancer; however, it is not a definitive diagnosis that determines treatment [10,11].

Considering the slow course of the disease and the older typical age of the patient, the treatment of prostate cancer must take into account the general well-being of the patient, the presence of comorbidities, and the expected quality of life. A low degree of local advancement along with a favorable histopathological diagnosis with a Gleason score < 7 and PSA < 10 indicates that the patient should be offered deferred treatment. The aim of such a procedure is to avoid the phenomenon of overtreatment, i.e., the introduction of therapy that is not necessary (does not save the patient’s life) and which may drastically reduce their quality of life [12]. Deferred treatment can take the form of active surveillance (AS) or watchful waiting (WW). The first method consists of postponing treatment until the disease progresses. Patients qualified for this method of treatment must be fully aware and agree to postpone the implementation of more radical treatment [13,14]. Radical treatment of prostate cancer includes, first of all, prostatectomy, i.e., surgical excision of the prostate involved in the neoplastic process. Radical prostatectomy is currently considered the gold standard of treatment for organ-confined disease, while hormonal treatment, immunotherapy, and chemotherapy bring only a palliative effect [15].

Scientific reports indicate the possibility of inhibiting, delaying, or reversing the process of carcinogenesis by consuming natural or synthetic phytopharmaceuticals. Substances of natural origin are a valuable source of minerals, vitamins, and antioxidants, which grant them a functional health-promoting character [16]. For a long time, oncologists have been conducting research on the effectiveness of taking natural/plant products in order to improve patient survival and comfort of life. Literature reports suggest that vegetables, herbs, and their extracts or derivatives may play an important role in the prevention or combined treatment of cancer [17,18].

Propolis is a natural sticky resinous substance produced by worker bees from the juices, resins, and exudates they collect from the buds, leaves, flowers, stems, and bark of many of the flowers and trees they visit. It is then used as a building block to repair and seal breaches in the hive, maintaining a relatively constant temperature and humidity. The composition of propolis changes depending on the hive, its location, and the season. It contains over 300 different identified compounds, including, among others: plant resins (50–80%), waxes (8–30%), polyphenols (14–16%), and pollen (5%) [19,20].

Flavonoids are secondary plant metabolites. To date, over 10,000 flavonoid compounds have been isolated and identified [21]. The best described property of almost every group of flavonoids is their antioxidant activity. In addition, they are effective antimicrobial substances against a wide range of microorganisms. Strong bacteriostatic and bactericidal properties have been confirmed for apigenin, galangin, flavone and flavonol glycosides, isoflavones, flavanones, and chalcones [22,23]. Flavonoids are characterized by potent antiviral activity as well. Quercetin, rutin, naringenin, apigenin, and hesperidin, reduce the infectivity of viruses by inhibiting the activity of enzymes necessary for their replication. Administered together with antiviral drugs, they increase their effectiveness and alleviate side effects. The effectiveness of flavonoids has been demonstrated against hepatitis, AIDS, flu, herpes, and SARS-CoV-2. The COVID-19 pandemic, which is an important health problem, has clearly shown that it is necessary to constantly search for new methods of safe treatment [24,25,26]. Meanwhile, certain flavonoids significantly affect the functioning of the immune system and inflammatory cells. A number of flavonoids, such as apigenin, hesperidin, luteolin, and quercetin, have analgesic and anti-inflammatory effects. It has been found that flavonoids are able to inhibit the expression and synthases of nitric oxide, cyclooxygenases, and lipoxygenases, which are responsible for the production of large amounts of nitric oxide, prostanoids, leukotrienes, and other mediators of the inflammatory process, such as chemokines, cytokines, or adhesion molecules [27,28].

Curcumin (CUR) is a yellow dye isolated from the root of *Curcuma longa* L. Numerous scientific studies have indicated that curcumin can be used in both anticancer chemoprevention and in support of cancer treatment. It has been found that curcumin can inhibit the development of cancer, e.g., by enhancing apoptosis in cancer cells and inhibiting metastases. Data from the literature confirm reports of antioxidant, immunosuppressive, and anti-mutagenic properties of curcumin [29,30,31]. Curcumin sensitizes cancer cells to chemotherapy and radiotherapy, has a protective effect on healthy tissues, and is not toxic to humans even in high concentrations [32]. Curcumin can regulate the activity of the transcription factor NF-κB (nuclear factor kappa-light chain enhancer of activated B cells), which is involved in the activation of pro-inflammatory cytokines, increase in the concentration of prostaglandins, and mobilization of immune system cells [33,34]. Particularly noteworthy is the ability of curcumin to sensitize cancer cells to the effects of chemotherapeutic drugs. Multidrug resistance on the part of cancer is a serious therapeutic problem conditioned by the activity of membrane proteins from the ABC family. By removing xenobiotics from cancer cells, ABC transporters force the use of higher drug doses, which in addition to damaging the cancer tissue may cause strong toxic effects in noncancerous cells. As a competitive inhibitor, curcumin blocks ABC protein receptors and facilitates the diffusion of drugs into cancer cells [35].

The main sources of quercetin (QUE) are grapes, blueberries, tomatoes, red wine, and black tea. It can be found in propolis as well. The anticancer, anti-inflammatory and antiviral effects of quercetin have already been proven, though further research is needed to explain its role in inhibiting lipid peroxidation, platelet aggregation, and capillary permeability [36,37,38]. Experiments using various cell lines have shown that quercetin inhibits lipopolysaccharide (LPS)-induced production of tumor necrosis factor α (TNF-α) and IL-8 in macrophages. In addition, it reduces the concentration of enzymes that cause inflammation (cyclooxygenases (COX) and lipoxygenases (LOX)) and inhibits the action of metalloproteinases, preventing modification of the environment of cancerous tumors and their metastasis. Particularly interesting is the apparent ability of quercetin to inhibit the cell cycle, reduce viability, inhibit proliferation in cancer cells, and induce apoptosis of altered cells [39,40]. Studies have shown that the administration of quercetin can reduce the expression of androgen receptors (AR) on the surface of prostate cancer cells, which can be widely used in hormone therapy for this cancer [41,42,43].

Caffeic acid phenethyl ester (CAPE) is naturally rare; the highest known CAPE concentrations have been detected in propolis. As a derivative of caffeic acid, CAPE is characterized by strong antioxidant properties. The antioxidant and anti-inflammatory properties of CAPE make it a compound with a wide range of biological activities. CAPE has been shown to have neuroprotective, hepatoprotective, and cardioprotective activities, and can be used in the prevention of cancer [44,45]. CAPE minimizes the adverse effects of certain anti-cancer drugs. The simultaneous use of CAPE and synthetic chemotherapeutics inhibits the formation of free radicals, thereby reducing the toxicity induced by, among others, doxorubicin, cisplatin, methotrexate, and tamoxifen. What is more, CAPE may increase the effectiveness of chemotherapy by sensitizing cancer cells to drugs. The antiproliferative and cytotoxic effects of CAPE have been documented in many cancer models, including on cell lines of colon cancer, lung cancer, melanoma, glioblastoma, pancreatic cancer, gastric cancer, biliary tract cancer, and breast cancer [46,47,48].

The direct aim of the present study is to evaluate the cytotoxic, anti-proliferative, and pro-apoptotic properties of selected flavonoids on prostate cancer cells of the LNCaP line. The goal of our experiments is to assess whether quercetin, curcumin, and CAPE inhibit the growth of prostate cancer cells and initiate the process of programmed death in these cells. The respective anticancer properties of the tested compounds are compared, and it is determined whether the cytotoxic effects of the tested compounds are dependent on the dose and time of intoxication of the cell line.

## 2. Results

### 2.1. XTT Test (Mitochondrial Activity)

#### 2.1.1. CAPE

The reduction of mitochondrial activity was most observed 24 h after the application of CAPE at concentrations of 10 and 25 μM. The other concentrations tested had a negative effect on the mitochondrial activity of the tested cells, though the detected decrease in mitochondrial activity was less rapid. The cytotoxic activity of CAPE only partially increased with increasing concentration of the tested compound. However, no decrease mitochondrial activity was observed when increasing the duration of the compound’s action on the LNCaP line.

#### 2.1.2. Curcumin

A decrease in mitochondrial activity in the tested cell line after application of curcumin was already observed at a concentration of 10 μM. Each of the tested concentrations decreased mitochondrial activity by a statistically significant amount. Activity decreased proportionally to the dose used and the duration of the experiment. When extending the incubation time, it was observed that selected doses of curcumin reduced the activity of mitochondria in the LNCaP line by more than half.

#### 2.1.3. Quercetin

A significant reduction in mitochondrial activity in the LNCaP line was observed after the use of quercetin at a concentration of 10 μM. This was the lowest of the tested concentrations, and after 24 h of exposure it resulted in a decrease in mitochondrial activity by more than half. Higher concentrations of quercetin decreased mitochondrial activity as well. The cytotoxic activity of quercetin increased with increasing duration of action on the LNCaP line.

### 2.2. NR Test (Lysosomal Activity)

#### 2.2.1. CAPE

In this study, a decrease in lysosomal activity was observed in LNCaP cells exposed to CAPE. The cytotoxic effect was partly dependent on the dose of the test compound and proportional to the incubation time. The strongest cytotoxic effect was caused by CAPE at a concentration of 50 μM after 24 h of incubation and as little as 10 μM after extending the experiment to 48 h.

#### 2.2.2. Curcumin

A decrease in lysosomal activity was observed in LNCaP cells exposed to curcumin after 24 h at a concentration of 10 μM. It is noteworthy that after the use of curcumin at a concentration of 25 μM, after both 24 and 48 h lysosomal activity in the LNCaP cells increased, while at subsequent doses it significantly decreased. Extending the duration of the experiment slightly increased the cytotoxic effect of curcumin.

#### 2.2.3. Quercetin

There was a dose- and time-dependent decrease in lysosomal activity in LNCaP cells exposed to quercetin. A statistically significant cytotoxic effect was observed after the use of quercetin at a concentration of 10 μM. Another rapid decrease in lysosome activity was observed in the tested cell line after exposure to the test compound at a concentration of 50 μM.

### 2.3. SRB Test (Total Protein Synthesis)

#### 2.3.1. CAPE

In this study, we observed a decrease in total protein synthesis in LNCaP cells after incubation with CAPE, that was dependent on both the concentration of the tested compound and its incubation time. The strongest cytotoxic activity was determined for the CAPE concentration equal to 10 μM after 24 and 48 h of incubation. The decrease in cell viability was significant after the application of CAPE at concentrations of 25 and 50 μM.

#### 2.3.2. Curcumin

The cytotoxic activity of curcumin against prostate cancer cells was confirmed by assessing the decrease in total protein synthesis. At the lowest tested concentration, curcumin statistically significantly reduced the synthetic activity of LNCaP cells. After applying curcumin at concentrations of 50 μM and 100 μM, total protein synthesis in cells slightly increased at both times tested.

#### 2.3.3. Quercetin

Quercetin at a dose of 10 μM decreased cellular protein synthesis by a statistically significant amount at both times tested. During 24 h of incubation, higher concentrations of quercetin did not cause as rapid a decrease in synthetic activity in prostate cancer cells. At a concentration of 100 μM, even a slight increase in protein synthesis was observed. Extending the experiment time to 48 h statistically significantly increased the cytotoxic activity of quercetin. The greatest difference in the effect of the tested compound was demonstrated for the concentration of 100 μM.

**Figure 1:**
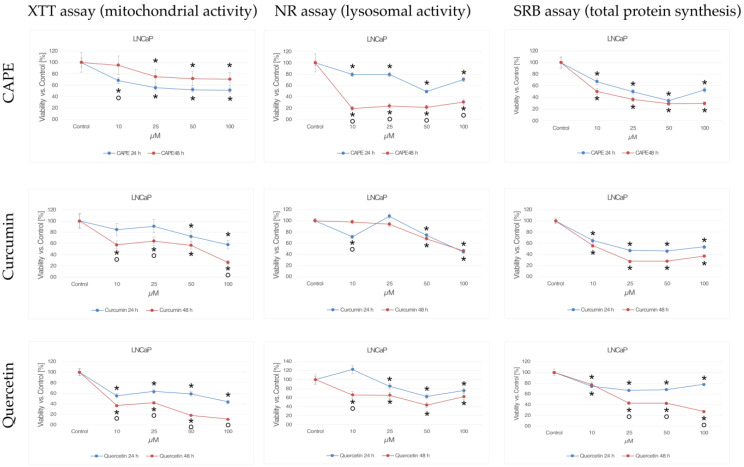
Cytotoxic effects of CAPE, curcumin, and quercetin at concentrations from 10 to 100 μM at 24 h and 48 h incubation time against the LNCaP prostate cancer cells using the XTT-NR-SRB assay. * statistically significant decrease in viability relative to control (*p* < 0.05); ° statistically significant difference in cell viability after extending tested compounds incubation from 24 to 48 h (*p* < 0.05).

Figure 1 shows the cytotoxic effect of CAPE, curcumin, and quercetin at concentrations of 10 to 100 μM at 24 h and 48 h incubation on the LNCaP prostate cancer cell line using XTT (mitochondrial activity), NR (mitochondrial lysosomal), and SRB (total protein synthesis).

### 2.4. IC50 Calculation

The IC50 value was calculated for the tested compounds using computer software (Table 1). The IC50 values indicate that the test compounds have cytotoxic activity against LNCaP prostate cancer cells.

Mitochondrial activity was most strongly inhibited by quercetin; 48 h incubation of the tested cells with quercetin allowed low doses of the compound to achieve high cytotoxic effectiveness. Prolonged incubation of LNCaP cells with curcumin had a statistically significantly effect, increasing the effectiveness of the compound. However, such a correlation was not observed for CAPE; the experiment showed that as prostate cancer cells were incubated with CAPE for a longer period, their mitochondrial activity increased.

Moreover, the activity of lysosomes was higher after extending the incubation time of the tested cells with CAPE. It was found that only higher concentrations of CAPE than those used in the experiment could inhibit the activity of lysosomes in half of the tested cells. An increase in lysosomal activity after extending the duration of the experiment was observed for quercetin as well. The strongest inhibition of lysosomal activity was calculated for curcumin. However, the cytotoxic activity of this compound did not show a statistically significant increase over time.

Quercetin inhibited cellular protein synthesis the most. It is noteworthy that very low concentrations of quercetin can inhibit protein synthesis in a short incubation time. It was observed that extending the time of the experiment had a negative effect on the cytotoxic activity of quercetin as expressed by the inhibition of the synthesizing activity of prostate cancer cells; in turn, exposure of the tested cells to CAPE and curcumin for 48 h increased the effectiveness of the compounds.

### 2.5. Mitochondria Activity Labeling with JC-1 Reagent

**Figure 2:**
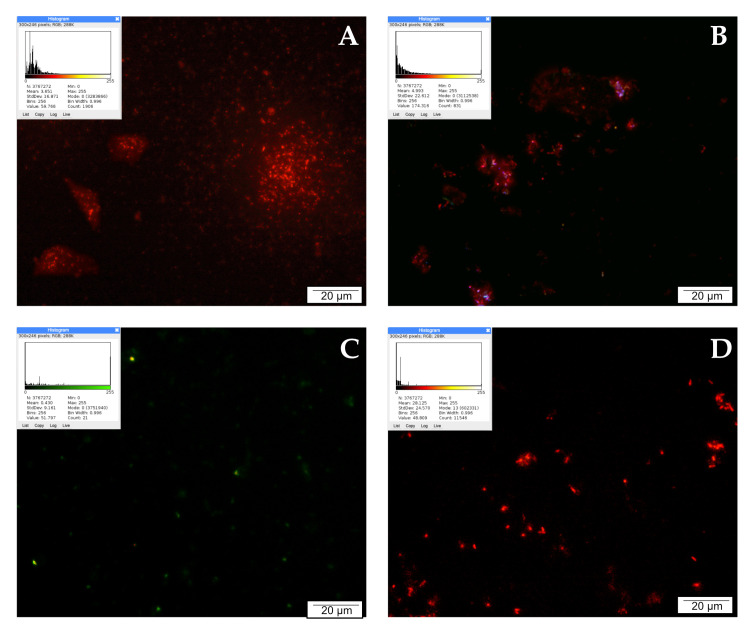
Inner mitochondrial membrane potential (ΔΨm): (**A**) cells from the control group, (**B**) cells treated with CAPE, (**C**) cells treated with CUR, and (**D**) cells treated with QUE. Quantitate intensity of wave response (red/green, respectively) represented by mean number (*p* < 0.05). Analyzed using ImageJ software, v. 1.53 m.

Mitochondria are involved in the process of cellular respiration, as a result of which the cell obtains energy. In addition, mitochondria regulate many other intracellular processes, such as cell signaling, maturation, differentiation, cell growth and death, and control of the cell cycle. JC-1 staining was used to assess mitochondrial activity in LNCaP cells, the results of which are shown in Figure 2. In the control sample, numerous bright red signals emitted by intact mitochondria are visible. Single green points observed in the microscopic image come from mitochondria with a depolarized membrane, and indicate cell damage/death.

After 24 h of incubation of the examined cells with CAPE at a concentration of 25 µM, high mitochondrial activity continued to be observed. In the presented documentation, intense red signals emitted by integral mitochondria are noticeable. The blue and green signals observed in the field of view may come from cells that naturally died during culture. CAPE did not damage the mitochondria, and even increased their activity. On the other hand, an antagonistic effect was demonstrated for curcumin. Prostate cancer cells exposed to curcumin for 24 h showed noticeably decreased mitochondrial activity. The microscopic images are dominated by green signals corresponding to damaged mitochondria. Single green signals were observed after treating the tested line with quercetin. In the microscopic image, however, mainly red moments are visible, suggesting that in these cells the mitochondria were not damaged and their activity remained at a high level.

### 2.6. Lysosome and Autolysosome Labeling with LysoTracker Red DND-99 Staining

LysoTracker Red DND allows red labeling of lysosomes with increased activity. Lysosomes are small irregular vesicles surrounded by a single protein–lipid membrane. They occur in the cytoplasm of the cell, and are primarily responsible for the digestion of substances that are unnecessary or harmful to the cell. The activity of lysosomal enzymes (proteases, nucleases, glycosidases, phosphatases, and lipases) is essential in the degradation process of macromolecules delivered to lysosomes, i.e., proteins, nucleic acids, sugars, and lipids. The control shows single red signals, most likely from activated lysosomes in damaged/dying cells. Increased activity of degrading enzymes may accompany autophagy or other types of cell death. However, the image is dominated by intensely green moments, which indicate a high percentage of living cells.

**Figure 3:**
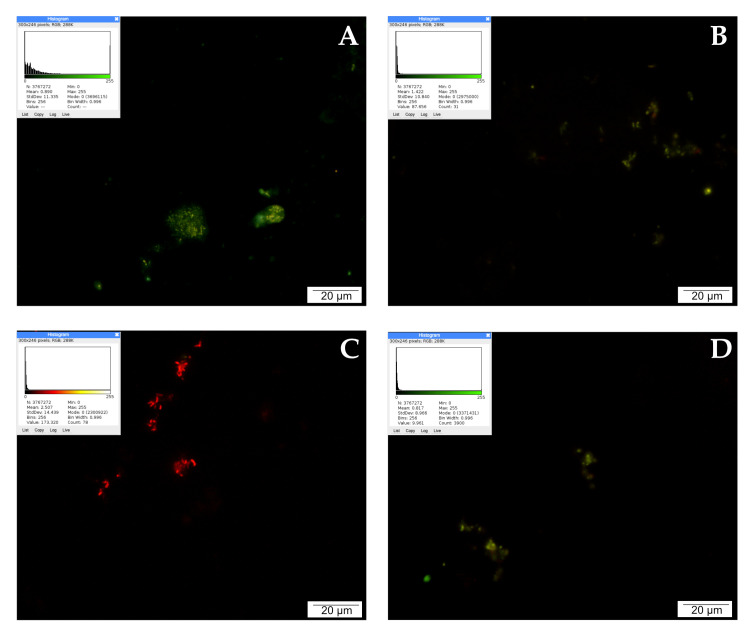
Lysosomal activity demonstrated by LysoTracker Red: (**A**) cells from the control group, (**B**) cells treated with CAPE, (**C**) cells treated with CUR, and (**D**) cells treated with QUE. Quantitate intensity of wave response (red/green respectively) represented by mean number (*p* < 0.05). Analyzed by ImageJ software, v. 1.53 m.

The highest intensity of red signals was observed after treating LNCaP cells with curcumin. The tested compound noticeably increased the number of active lysosomes compared to the control. The intensely glowing lysosomes were clustered around the cell nuclei. Quercetin affected the activity of lysosomes as well; 24 h incubation of prostate cancer cells with a low concentration of 25 µM quercetin intensified lysosome activity, which is visible in the microscopic image as the presence of several bright red and orange signals. The effect of CAPE on lysosome activity was rather weak. No multiplication of red signals can be observed in the microscopic image, as the examined cells were poorly stained. The described results are presented in Figure 3.

### 2.7. Detection of Apoptotic Cells by the TUNEL Method

When detecting apoptosis using the TUNEL method, it is important to take into account that fragmentation of DNA into segments equal to multiples of the length of nucleosomes occurs in the cell during programmed cell death. Terminally deoxynucleotidyl transferase (TdT)-labeled dUTP-biotin cells that undergo apoptosis appear as intense red signals under a fluorescence microscope. Single red signals recorded in the control testify to the programmed death that occurs physiologically during cell culture as a result of fluctuations in temperature, pH, or nutrient concentrations.

**Figure 4:**
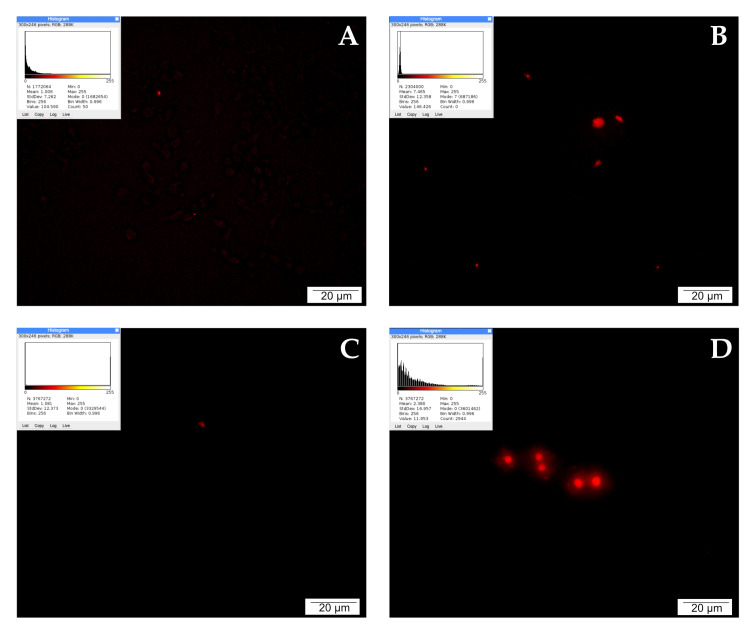
Apoptosis demonstrated by the TUNEL method: (**A**) cells from the control group, (**B**) cells treated with CAPE, (**C**) cells treated with CUR, and (**D**) cells treated with QUE. Quantitate intensity of wave response (red/green respectively) represented by mean number (*p* < 0.05). Analyzed by ImageJ software, v. 1.53 m.

LNCaP cells treated with CAPE were stained intensely red. A large accumulation of signals visible in the microscopic image may suggest the initiation of apoptosis by the tested compound. Weaker events were recorded in the culture treated with curcumin. The picture of the cells is non-specific, and the number of red signals is comparable to the control. After 24 h of incubation of the tested cells with quercetin, a very strong intensification of red signals was observed. The cells were stained bright red, with large centrally located nuclei and a fairly abundant rim of granular cytoplasm. The described results are presented in Figure 4.

### 2.8. Detection of Reactive Oxygen Species (ROS)

**Figure 5:**
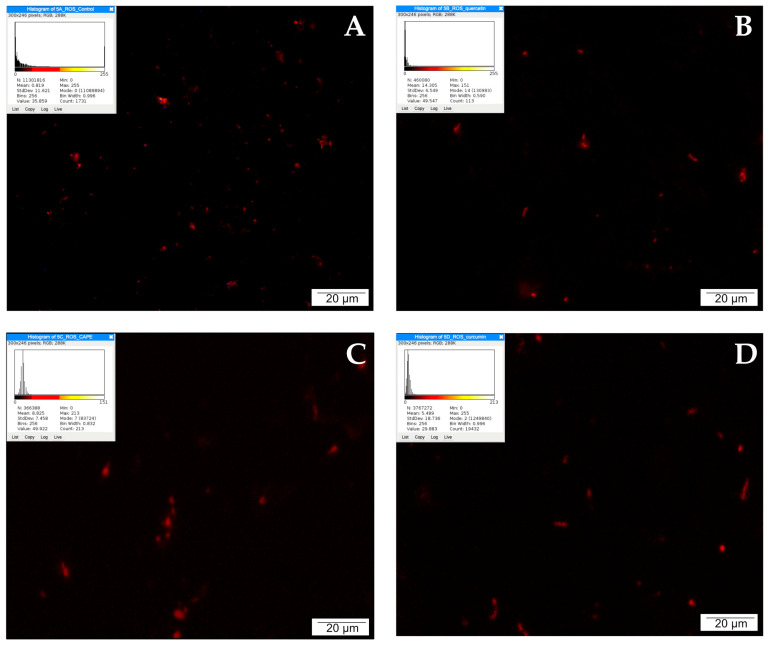
The presence of ROS demonstrated with the participation of dihydroethidium (DHE): (**A**) cells from the control group, (**B**) cells treated with quercetin (QUE), (**C**) cells treated with CAPE, and (**D**) cells treated with curcumin (CUR). Quantitate intensity of wave response (red/green respectively) represented by mean number (*p* < 0.05). Analyzed by ImageJ software, v. 1.53 m.

The LNCaP cells were treated with 25 µM of each of quercetin, CAPE, and curcumin, and were additionally stained for the presence of ROS. All compounds showed multiple red signals after incubation for 24 h, in the following order: quercetin > CAPE > curcumin > control. These results clearly indicate the initiation of the process of ROS release, with quercetin providing the most notable advantage. The obtained results are presented in Figure 5.

### 2.9. Transmission Electron Microscopy

With the invention of the transmission electron microscope, electron microscopy has become a key technique that allows cellular structures to be observed at the atomic level. In the TEM technique, the electron beam that passes through the sample allows imaging of structures up to 1 micrometer thick. In our experiments, structural changes in LNCaP cells treated with CAPE, curcumin, and quercetin at a concentration of 25 µM were studied.

Prostate cancer cells in the control group showed a structure typical of cancer cells. Numerous microvilli could be seen on the surface of their integral cell membrane, probably enabling intercellular contact and stabilizing the cells in the substrate. Numerous undamaged mitochondria and fragments of the rough endoplasmic reticulum together with dictyosomes were visible in the cytoplasm. The presence of individual lysosomes was observed as well.

**Figure 6:**
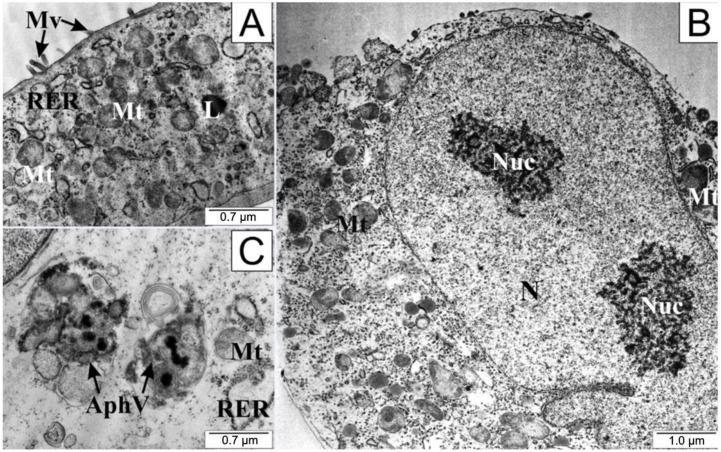
Electronograms of LNCaP cells: (**A**) from the control group and (**B**,**C**) treated with CAPE. AphV—autophagosomes, L—lysosome, Mt—mitochondria, Mv—microvilli, N—cell nucleus, Nuc—nucleoli, RER—rough endoplasmic reticulum.

After the application of 25 µM CAPE in the test cells, a definite folding of the cell membrane, shortening or complete disappearance of cytoplasmic extensions, and protrusion of the cell nucleus were observed (Figure 6). Nucleoli visible in the nucleus had a loose structure. On the other hand, numerous undamaged mitochondria could be seen in the cytoplasm, accompanied by the formation of autophagosomes. Autophagosomes are formed by separating a fragment of the membrane from the endoplasmic reticulum, which divides the contents of the cell cytosol with organelles into compartments. After fusion of the autophagosome with the lysosomes, the contents of the resulting vesicle are digested using lysosomal enzymes.

**Figure 7:**
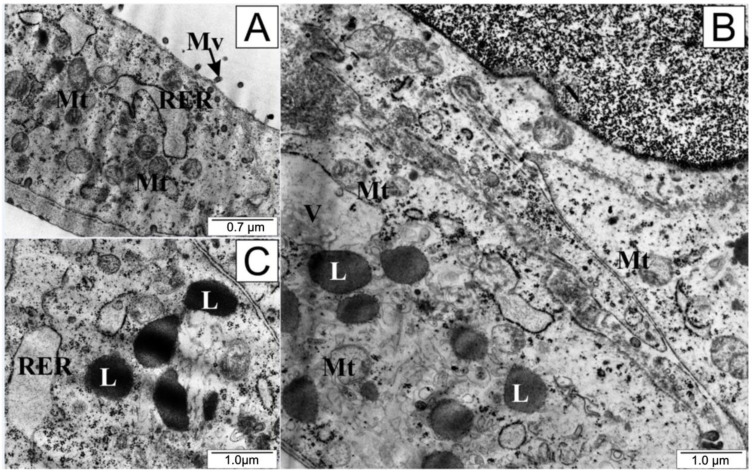
Ultrastructure of LNCaP cells: (**A**) from the control group and (**B**,**C**) treated with curcumin. L—lysosomes, Mt—mitochondria, Mv—microvilli, RER—rough endoplasmic reticulum, V—vacuoles.

Cells treated with curcumin presented a definitively different microscopic picture (Figure 7); they were swollen, and their plasma membranes were torn. Notably, there was an accumulation of numerous granules and vacuoles inside the cytoplasm, along with disintegration of elements of the endoplasmic reticulum and mitochondria. Numerous swollen lysosomes were observed in the cytoplasm. In the cell nucleus, chromatin concentration was visible.

**Figure 8:**
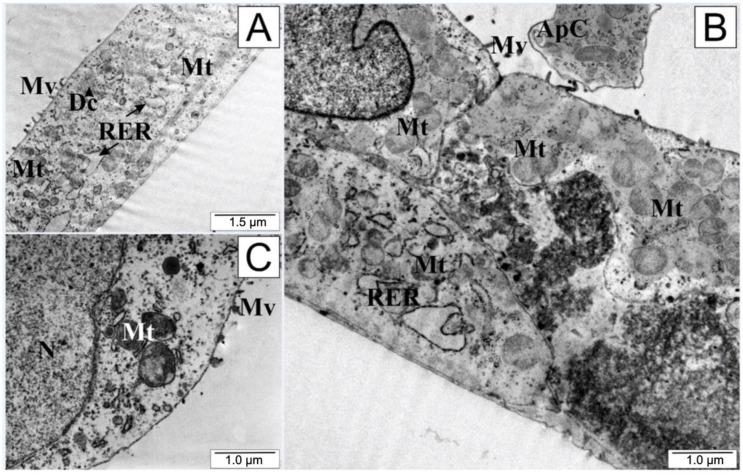
Electronograms of LNCaP cells: (**A**) from the control group and (**B**,**C**) treated with quercetin. ApC—apoptotic body, Dc—dictiosome, Mt—mitochondria, Mv—microvilli, N—cell nucleus, RER—rough endoplasmic reticulum.

An interesting microscopic picture (Figure 8) was observed after treating the tested cells with a low dose of quercetin. The integrity of the cell membranes was preserved, although there was marked shortening of the cytoplasmic protrusions, suggesting that the cell separated from both the environment and from the other cultured cells. The endoplasmic reticulum and mitochondria retained their shape and size. In the cytoplasm, the presence of cellular organelles surrounded by cell membrane could be observed. A bulging cell nucleus with condensed chromatin (especially visible in Figure 8B) and the formation of apoptotic vesicles was characteristic of quercetin exposure.

## 3. Discussion

Cancer is one of the biggest problems in modern medicine. Prostate cancer is the third most frequently diagnosed malignant tumor in the world, and its incidence is increasing [49]. Most men develop benign prostatic hyperplasia as they age. In most cases, benign prostatic hyperplasia has no serious clinical symptoms apart from voiding disorders. Only late symptoms of malignant growth, which may include fatigue due to anemia, bone pain, and kidney failure, direct the patient to intensive diagnostics, often resulting in late treatment [50].

Early detection of prostate cancer has a good prognosis. The therapeutic regimen for prostate cancer, which has been revolutionized over the last decade thanks to new treatment methods, primarily involves the surgical removal of this organ. It is worth emphasizing that more and more often prostate excision procedures are performed using robotic surgery. However, taking into account not only the stage of the cancer but the patient’s age, comorbidities, and molecular characteristics of the lesion, surgical treatment is often associated with brachytherapy (local radiotherapy), teletherapy (external radiotherapy), and/or hormonal antiandrogen treatment (ADT) [51,52].

Alleviating the side effects of treatment can be achieved by reducing the effective dose of the chemotherapeutic drug or simultaneous administration of chemotherapeutics with substances showing cytotoxic and pro-apoptotic properties. Research is underway to determine whether compounds of natural origin could be used in anticancer therapy.

Flavonoids have proven anti-inflammatory, immunomodulatory, and antioxidant activity. They are used in the treatment of infections and hard-to-heal wounds and supportive in the prevention of cancer. CAPE has been shown in many experimental studies to have the ability to eliminate reactive oxygen species and synthetic radicals, as well as to chelate metal ions and inhibit lipid peroxidation [53,54]. In addition, the pro-apoptotic effect of CAPE by modulation of the NF-kappaβ pathways and changes in the cell cycle resulting from the activity of the p53 protein has been proven. Studies have shown that CAPE binds to the Fas death receptors on the surface of cells and activates two paths of apoptosis initiation, FADD/caspase-8 and JNK/p38 [55]. Activation of the first of these pathways leads to an increase in the activity of executive caspases 3 and 7. In the second mechanism, mitogen-activated protein kinases (MAPK) are stimulated. Both JNK (c-Jun N-terminal kinase) and p38 MAPK regulate the activity of many proteins, enzymes, and transcription factors; thus, they may limit the proliferative potential of cells, regulate their cell cycle, and induce apoptosis [56]. Increased activity of pro-apoptotic genes (BAD, CASP8, FAS, FADD, p53) can be observed in cancer cells treated with CAPE [57,58]. The use of CAPE induces the rapid loss of mitochondrial transmembrane potential, release of cytochrome c from the mitochondrial intermembrane space (IMS), formation of the apoptosome, and the activation of caspases. Mitochondria are organelles in which apoptotic signals from various pathways are integrated [59]. Reducing the proliferation and migration of cancer cells due to the effect of CAPE on cytoskeletal proteins and spindle function has been demonstrated, along with related changes in the tumor microenvironment [60]. Quercetin, in turn, stops cancer cells in the G0/G1 phase of the cell cycle, promotes apoptosis, and reduces angiogenesis by interfering with the PI3K/AKT/mTOR and STAT3 pathways. Reducing the expression of anti-apoptotic proteins such as c-FLIP, cyclin D1, and cMyc allows for more effective elimination of altered cells [61,62,63]. In turn, lower concentrations of pro-inflammatory IL-1β, IL-6, and TNF-α inhibit neoplastic proliferation and formation of new blood vessels. It has been shown that quercetin significantly increases the activity of caspases, initiating programmed cell death [64]. Similar molecular pathways are affected by curcumin. Its anti-cancer properties are based on antioxidant activity (lowering the concentration of ROS, catalase, and glutathione peroxidase) and anti-inflammatory activity related to the influence on the transcription factor NFkappaβ and Notch-1 [65,66]. The important role of curcumin in relation to the ability of cancer cells to actively remove cytotoxic drugs has been described as well. Inhibition of the activity of cytochrome P-450 and its reductase by curcumin allows for higher concentrations of cytotoxic substances in cells [67].

The direct aim of this study was to evaluate the effect of selected flavonoids on prostate cancer cells of the LNCaP line in in vitro studies. The effects of the administration of CAPE, quercetin, and curcumin to LNCaP cells on the mitochondrial and lysosomal activity and the total synthesis of cellular proteins were examined, and IC50 values for the tested compounds were determined. In addition, mitochondrial and lysosomal activity and the presence of apoptotic cells were determined using immunofluorescence techniques. Due to promising results, an additional assay of reactive oxygen species was performed for quercetin.

As described in the literature, the morphology of non-intoxicated prostate cancer cells of the LNCaP line is typical of cancer cells. The examined cells have an elongated spindle shape and form long cytoplasmic protrusions. The cytoplasm is rather poor and grainless, and in certain cells the presence of small vacuoles is visible at higher magnifications. The cells are dominated by a single large and darkly-stained nucleus. Chromatin is unraveled and nucleoli are visible in the nuclei, which suggests high proliferative potential on the part of the LNCaP cells [68,69]. After observing the morphology of the examined cells in an inverted microscope and via screening staining with hematoxylin and eosin, we performed imaging of the LNCaP line with a transmission electron microscope. It was observed that each of the tested compounds had different effects on LNCaP cells.

After CAPE treatment, the prostate cancer cells shortened their cytoplasmic protrusions and thickened their cytoplasm, while their inner membranes remained integral. While numerous mitochondria were visible in the cells, they formed autophagosomes as well, suggesting that the autophagy process was initiated in the cells. A characteristic feature of autophagy is initially selective elimination of cell organelles damaged by toxic substances, followed later by the disintegration of the entire cell. Thanks to this process, toxins or pathogens accumulated in cells can be removed. Autophagy can be observed in cells deprived of access to nutrients, in which case digested protein substances become a source of the amino acids necessary to maintain basic cellular metabolism [70,71,72].

Transmission electron microscopy was the first method of observing autophagy, and it remains the most reliable and sensitive method of observation [73]. In the work of Chang et al., it was noted that CAPE derived from Chinese propolis significantly inhibited LPS-stimulated MDA-MB-231 cell proliferation, migration, and production of NO. It has been shown that CAPE can induce the process of autophagy by increasing the activity of LC3-II and decreasing p62 levels. LC3 is the most widely used marker of emerging autophagosomes [74]. An increase in the activity of this protein has been observed in the SH-SY5Y human neuroblastoma cell line when treated with CAPE [75]. In C6 glioma cells, autophagy was stimulated by activation of AMPK (5’AMP-activated protein kinase [76,77]. Additionally, CAPE derivatives have been found to induce autophagy, e.g., in a colon cancer cell line [78].

The morphology of LNCaP cells treated with curcumin would suggest necrosis. This is evidenced primarily by the swelling of the cell and its organelles caused by unsealing of the cell membrane and the influx of ions and water into the cell. Numerous vacuoles and lysosomes visible in the cytoplasm suggest an intensification of the decomposition of substances released from the disintegrated structures of the endoplasmic reticulum and mitochondria. Meanwhile, the literature is dominated by reports that curcumin is a compound that induces apoptosis in cancer cells. Many studies indicate that curcumin causes the activation of programmed cell death in cancerous cells by reducing the concentration of TNF-α and survivin, regulating the activation pathway of mitochondrial caspases, inhibiting the PI3-Kinase/AKT pathway, and increasing the expression of the BAX gene, [79,80,81]. A study by Li et.al showed that curcumin may promote autophagy in gastric cancer cells through a process characterized by the formation of acidic vesicular organelles (AVOs), conversion of LC3-I to LC3-II, and an increase in the levels of autophagy-related proteins [82]. Changes in the morphology of curcumin-treated cells observed in electron microscopy have been described in Perrone et al., among other studies. These studies point out that compounds of natural origin may have a preventive effect in neurological and neuromuscular diseases by inducing autophagy in cells with damaged organelles. Curcumin can affect each of three types of autophagy: macroautophagy, microautophagy, and chaperone-mediated autophagy. It induces the formation of the phagophore and the fusion of the outer membrane of the autophagosome with the lysosome [83]. Moreover, an assessment of the effect of curcumin on inflammatory bowel disease in mice showed that the tested compound enhances autophagy in intestinal epithelial cells. More vacuolar structures of bilayer were observed under microscope, and were found to contain cytoplasmic components such as mitochondria [84]. A new curcumin analog developed by Zhou et al., -3,5-bis(2-hydroxybenzylidene)tetrahydro-4H-pyran-4-one glutathione conjugate (EF25-(GSH)2), has shown the ability to induce autophagy in cancerous cells. In three human hepatocellular carcinoma cell lines (HepG2, SMMC-7721 and BEL-7402) and one immortalized human hepatic cell line (HL-7702), it showed apparent vacuolization in the cytoplasm after the application of 5 µmol/L EF25-(GSH)2 in a 16 h experiment. The use of higher doses of this curcumin derivative resulted in the appearance of large vacuoles of varying size which were content-free and single-membrane bounded, while smaller vacuoles resembled autophagic vacuoles. In addition, multimembrane autophagic vesicles engulfing cytoplasmic components and organelles were identified in the cytoplasm [85].

Zhang et al., while conducting research on the murine hippocampal cell line HT-22, showed that curcumin can inhibit neurocyte autophagy. The neuroprotective effect of curcumin is based on its antioxidant properties, and inhibits amyloid aggregation (Aβ, especially Aβ-42); when a cell is free of pathological deposits, this inhibits the autophagy process [86].

The difference in the results of our experiment may be due to the dose of curcumin selected for testing (in the cited experiments, the concentration was not higher than 10 μM), the duration of the experiment, or the particular sensitivity of prostate cancer cells to this compound. Different types of cell death may be interdependent. There is cross-talk between the autophagy and apoptosis pathways. In human cells, the protein linking the processes of apoptosis and autophagy is beclin 1, which is accompanied by number of caspases and the MGB1 protein (high mobility group box 1) [87]. Researchers have described the phenomenon of programmed necrosis. Necroptosis can be induced by specific ligand activation of several cell membrane receptors (CD95 (including FAS), TNFR1, TNFR2, TRAIL1, and TRAIL2) as a result of ATP depletion and oxidative stress [88,89]. In the course of necroptosis, plasma membrane rupture, cell swelling, and cell rupture are observed. Furthermore, necroptosis is characterized by an increase in cell volume, perforation of the plasma membrane, cellular collapse, and release of cellular contents [90,91]. It is possible that, in our experiment, cells of the LNCaP line underwent a process of programmed necrosis under the influence of curcumin; however, confirmation of this thesis requires further research.

Quercetin directed the tested cells into the programmed death pathway, i.e., apoptosis. Forming apoptotic bodies, gradual invagination of the outer cell membrane, and compaction and fragmentation of the cell nucleus are typical morphological features of apoptotic cells. Quercetin targets apoptosis by upregulating Bax, caspase-3, and p21 while downregulating Akt, PLK-1, cyclin-B1, cyclin-A, CDC-2, CDK-2, and Bcl-2 which has been widely discussed in the literature [92,93]. Moreover, reports have suggested that the anti-diabetic, hepatoprotective, and cardioprotective properties of quercetin may be related to the activation of autophagy processes [94]. In the example of the lung cancer cell lines A549 and H1299, it has been shown that programmed death may be preceded by autophagy processes regulated by the SIRT1/AMPK pathway [95].

Fluorescence techniques were used in our experiments to determine the effect of the tested compounds on the activity of mitochondria and lysosomes and the initiation of apoptosis processes. The method using the JC-1 reagent showed that the mitochondrial activity of LNCaP cells exposed to CAPE was comparable to the intensity of signals obtained from the mitochondria of cells in the control system. Curcumin administration visibly decreased mitochondrial activity, while a subtle intensification of signals from undamaged mitochondria was observed in prostate cancer cells exposed to 25 µM quercetin.

Labeling of lysosomes and autolysosomes with LysoTracker Red DND-99 revealed intense red signals from prostate cancer cells, which were apparently necrotic under the influence of the curcumin administered for culture. Cells treated with both CAPE and quercetin showed only single red signals, which was comparable to the cells in the control group.

Cells undergoing apoptosis were observed through the TUNEL technique. Single red signals emanating from apoptotic cells in the control suggest physiological elimination of worn-out or damaged cells. These signals are visible in the images of cells cultured with 25 µM CAPE and curcumin. The strongest red signals were recorded for LNCaP cells exposed to quercetin. Due to the predicted strong apoptotic activity of quercetin, in the next stage of the experiment we decided to stain the cells exposed to this compound for the presence of ROS as well.

Reactive oxygen species (ROS) are among the most important mutagens that occur naturally in the body, and cause genetic instability in cells. Such instabilities can generate mutations (from the completely harmless to the lethal) that lead to the elimination of the cell or its neoplastic transformation. Many biochemical reactions in which oxygen is metabolized can lead to the formation of toxic reactive intermediates that damage DNA [96].

The metabolic changes associated with carcinogenesis contribute to a high degree of oxidative stress in the tumor environment. However, defense against oxidative stress that allows cancer cells to survive is one of their metabolic features [97,98]. This is due to the fact that most cancer cells no longer use total oxidative phosphorylation to generate ATP, instead relying on glycolysis (the so-called Warburg effect) [99]. Such an effect has a significant impact on the energy supply of cells as well as on the redox system, immunity, and tumor adaptation [100]. Studies have indicated the primary role of mitochondria in the initiation and regulation of apoptosis. Oxidative stress is one of many factors that can cause unsealing of the mitochondrial membranes and the outflow of calcium ions from inside the mitochondria, which precedes other morphological changes characteristic of apoptosis, i.e., chromatin condensation, DNA fragmentation, and changes in the outer cell membrane [101,102]. This can lead to the formation of large amounts of reactive oxygen species. Our experiment using 6-carboxy-2’7’-dichlorofluorescein diacetate showed an increase in the number of red signals in the culture treated with quercetin in relation to the control group.

Our experiments additionally sought to determine which type of cell death occurs in prostate cancer cells intoxicated with selected flavonoids. The most beneficial type of cell death seems to be apoptosis, through which the body regulates, among other things, the processes of growth, maturation, and elimination of worn-out or diseased cells. Apoptosis is a natural phenomenon in human development and life. In contrast to necrosis, inflammation does not develop in the course of apoptosis, the molecular basis of which is similar to the changes observed in neoplastic tissues [103,104].

The morphology of apoptotic cells is definitely different from that of necrotic cells. The changes observed in the cell are organized and self-limiting, with nuclear chromatin condensation and DNA fragmentation into fragments of about 180 base pairs and their multiples. The cytoskeleton disintegrates, and the cell lengthens and loses contact with the surrounding microenvironment. Apoptotic bodies are formed which contain intact cellular organelles and are phagocytosed by phagocytic cells [105].

Numerous experiments have shown that chemotherapy eliminates cancer cells through a mechanism other than apoptosis. Studies have shown that the standard cancer treatment is based on the induction of autophagy, mitotic catastrophe, or necrosis. Autophagy, like apoptosis, is a planned and safe mechanism to eliminate cells in which abnormal proteins are deposited. Most often, it is activated by nutrient deficiencies, damage caused by external toxins, or cytokines that regulate development and differentiation. However, it has been shown that autophagy may increase the survival of cancer cells under stress conditions (radiotherapy, chemotherapy) by eliminating their damaged organelles [106,107,108,109]. Mitotic catastrophe does not have a clear definition; it is assumed that after cell damage and/or dysregulation of cell cycle checkpoints, chromosome aberrations and nuclear fragmentation occur and large cells containing one large nucleus are formed. Two possible mechanisms of mitotic catastrophe induction are observed in cancer cells. The first concerns cells that, undergo division immediately after the action of the damaging factor, which is not inhibited by proteins that control the cell cycle. The second type of mitotic catastrophe is observed in cancer cells entering the division cycle, and is preceded by the arrest of cell growth after the action of damaging factors [110,111].

Cells subjected to strong damaging stimuli die by necrosis. Necrosis is a pathological process leading to disruption of the cell membrane, a decrease in ATP levels, and destruction of the cell nucleus, endoplasmic reticulum, and lysosomes. Disintegrating cells release their contents into the environment, leading to the development of inflammation [112].

In our experiments, the viability of the examined cells was assessed by measuring their mitochondrial and lysosomal activity and the ability to complete the synthesis of cellular proteins. Using the XTT-NR-SRB triple test, we tested how the LNCaP line reacted to selected concentrations of CAPE, quercetin, and curcumin. The experiment showed that very low concentrations (10–25 µM) of the tested compounds inhibit the metabolism of cancer cells to a statistically significant extent. The strongest cytotoxic effect, measured by the assessment of mitochondrial activity, was demonstrated for quercetin. The inhibition of cellular protein synthesis decreased most significantly after treatment with quercetin. In turn, curcumin showed the strongest inhibition of the activity of lysosomes, while CAPE showed a strong effect on the synthesizing activity of the examined cells; this effect was the strongest on the first day of the experiment. Extending the duration of the experiment mainly intensified the anticancer properties of curcumin.

Quercetin has already been proven to have an effect on prostate cancer cells. The literature suggests that selected doses of quercetin can activate apoptosis in cancer cells. It has been shown that an increase in the sensitivity of prostate cancer cells to pro-apoptotic TRAIL ligands occurs after the application of 50–100 μmol/L quercetin. In addition, quercetin leads to activation of a caspase cascade, including caspase-3 and caspase-9, as well as to increased expression of the death receptor DR5 [37,42,113]. Quercetin enhances the apoptosis of prostate cancer cells by suppressing the synthesis of the main anti-apoptotic protein of the Akt family. The serine-threonine kinase Akt, known as protein kinase B (PKBP), plays one of the most important functions in cell proliferation, basic metabolism, and survival. Increased Akt activity is usually correlated with tumor progression and poor prognosis [114,115]. However, the most important role of quercetin in the induction of apoptosis in vitro is related to its effect on the concentration of proteins of the Bax/Bcl2 system. Lee et al. previously reported a rapid increase in Bax concentration on the surface of mitochondrial membranes in LNCaP cells. The application of 100 µM quercetin for 24 h caused the release of cytochrome from the transmembrane space of the mitochondria and its binding first to adapter proteins and then to procaspases. It is worth noting that the same (100 μM) concentrations of quercetin had no effect on viability or apoptosis in the normal human prostate epithelial cell line (PrEC) and the rat prostate-derived epithelial cell line (YPEN-1) [116,117]. Similar results have been shown for other cell lines as well. Experiments on MCF-7 breast cancer cells, HK1 nasopharyngeal cancer cells, SCC-9 oral cancer cells, and HL60 leukemic cells have confirmed the activation of the intracellular apoptosis activation pathway by quercetin [118,119,120,121,122].

Due to the particularly effectiveness of quercetin against cancer cells, there have been reports studying the possible inclusion of this compound in chemotherapeutic treatment. Studies by Zhang et al. have shown that the simultaneous administration of quercetin and paclitaxel to prostate cancer cells can inhibit cell proliferation, increase apoptosis, and induce cell cycle arrest at the G2/M stage. In addition, as demonstrated in our experiments as well, quercetin enhanced the synthesis of reactive oxygen species [123,124]. It is worth noting that quercetin has been found to reduce the resistance of cancer cells to docetaxel. Simultaneous administration of quercetin and docetaxel strongly weakened the activity of the PI3K/Akt pathway and allowed for the induction of programmed death [125].

Our experiments showed that the use of flavonoid derivatives reduces the viability of LNCaP prostate cancer cells, and is partly dependent on the dose and duration of the experiment. The type of cell death induced by the tested compounds was different. Accurate determination of the type of cell death requires extensive molecular research; however, based on morphological imaging, fluorescence, and immunoenzymatic methods, we are able to conclude that quercetin induces apoptosis in LNCaP cells. The effects of CAPE and curcumin remain debatable; most likely cancer cells begin the process of autophagy and/or necrosis under their influence.

The research conducted is this study requires continuation. It is necessary to clarify the molecular pathways with which the tested compounds interfere. Moreover, it is worthwhile to evaluate the effects of simultaneous administration of selected flavonoids and chemotherapeutic agents routinely used in the treatment of prostate cancer. It is possible that the additive cytotoxic effect of compounds of natural origin with paclitaxel or doscorubicin could allow the use of less aggressive doses of chemotherapeutics and alleviate the side effects of therapy. In addition, it is necessary to investigate whether effective doses of quercetin, CAPE, and curcumin can be introduced and maintained in the tumor microenvironment.

The possibility of using compounds of natural origin in the treatment of cancer is a great opportunity for modern medicine. However, the solution to the research problems caused by the bioavailability, multidirectional action, and the involvement of flavonoids in various metabolic processes requires further research.

## 4. Materials and Methods

### 4.1. Cell Coulturing

The experiments in this study were carried out using the LNCaP prostate cancer cell line from the ECACC (European Certificated Authorized Cell Culture) collection. Cells were cultured in RPMI-1640 medium supplemented with 5% serum. A mixture of antibiotics was added to the culture medium at the following concentrations: 100 IU/mL penicillin, 100 µL/mL streptomycin, and 0.25 µL/mL amphotericin B. Prostate cancer cell lines were cultured at 37 °C in an environment with 95% humidity and 5%CO_2_ concentration, which determined the maintenance of the correct pH of the culture medium. Passaging was performed with trypsin at a concentration of 0.25% in PBS (phosphate buffered saline) after reaching ~80% confluence. The time of cell adherence to the medium was estimated at 12 h. The experiment was performed at a cell density of 2.5 × 10^6^ cells/mL (counted in a hemocytometer).

The experiment used synthetic curcumin (Cat. No.: C7727), quercetin (Cat. No.: H5254) and CAPE (Cat. No.: 211200). The reagent supplier was Sigma-Aldrich, Poland. The test compounds were dissolved in DMSO (dimethylsulfoxide) to obtain a stock concentration of 5 mg/mL, then diluted in culture medium to obtain concentrations of 10, 25, 50, and 100 µM. The effects of DMSO on the tested cell line were assessed in a study preceding the experiment; no toxic effect of DMSO on LNCaP cells was found for the concentration used to dissolve the compounds.

### 4.2. Determination of the IC50 Minimum Inhibitory Concentration

IC50 values were determined for the LNCaP prostate cancer cell line exposed to the test compounds, i.e., the concentrations of the test compounds at which the viability of prostate cancer cells was inhibited in 50% of the culture. The IC50 value was calculated using ED50 plus software version 1.0 in the form of a Microsoft Excel spreadsheet for pharmacological analysis, allowing for the creation and analysis of concentration/response curves.

### 4.3. XTT-NR-SRB Triple Test

The XTT-SRB-NR test (Xenometrix; supplier: TIGRET Sp. z o.o., Warszawa, Poland; catalog number: PAN I 96.1200) uses three simple chemical reactions for the simultaneous determination of several parameters of cell viability. The reduction of tetrazolium salts (XTT) to formazan by the action of mitochondrial succinate dehydrogenase (an enzyme active only in cells with intact metabolism and respiratory chain) allows for the assessment of mitochondrial activity. Lysosomal activity is determined using the ability of NR (neutral red), a weak cationic dye, to cross the cell membrane and accumulate in lysosomes. Total cellular protein synthesis, on the other hand, is measured by the binding of sulforhodamine B (SRB) to proteins by weak electrostatic bonds. The assay was performed in six replicates in three independent experiments while strictly following the manufacturer’s instructions

### 4.4. JC-1 Staining (Mitochondria Activity Labeling)

JC-1 reagent was used to label active and inactive mitochondria. JC-1 is a cationic dye used as a fluorescent mitochondrial potential probe. This dye forms aggregates (fluorescent red) only in cells with intact mitochondria that have high membrane electrical potential. In the case of mitochondrial depolarization, JC-1 forms green fluorescent monomers.

The cells used for the experiment were seeded on six-well plates (CorningTM, Mediatech, Inc., Manassas, VG, USA); after reaching the appropriate confluence, they were treated with the test substances. The following concentrations were used in the study: 25 μM each of quercetin, curcumin, and CAPE. After 24 h of cultivation, the experiment was started. Viable cells were washed with PBS (3 × 5 min, 37 °C) and incubated with JC-1 dye (Invitrogen, Waltham, MA, USA) prepared in PBS for 20 min at 37 °C in the dark. After incubation, the cells were washed again in PBS (3 × 5 min) and stained with Hoechst 33342 (Life Technologies, Carlsbad, CA, USA) reagent (15 min, 37 °C, in the dark). The preparations prepared in this way were sealed in VECTASHIELD (Vector Laboratories, Newark, CA, USA) mounting medium and analyzed using an OLYMPUS BX60 microscope with appropriate filters. Micrographs were taken using an XC50 digital camera (Olympus, Tokyo, Japan). The image was developed in cellSens Standard computer software (Olympus, ver. 1.8.1). The results of the reaction were interpreted as follows: red/orange fluorescent signals indicate the high potential of the mitochondrial membrane and the preservation of its integrity, while the emission of green color signals suggests a decrease in the value of the mitochondrial membrane potential (i.e., the dye is present in the form of monomers). The quantitative evaluation of fluorescence was performed using ImageJ Software version 1.53 m (NIH, Bethesda, MD, USA). Each sample image was processed in a lookup table (LUT), with the red or green hot spots respectively used for lookup and higher mean values indicating higher intensity of the wave response.

### 4.5. LysoTracker Red DND-99 Staining (Lysosome and Autolysosome Labeling)

For the labeling of lysosomes and autolysosomes, staining was performed using LysoTracker Red DND-99 reagent, which is a fluorophore that selectively accumulates in lysosomes. LNCaP cells were reseeded in six-well plates (Corning™, Mediatech, Inc., Manassas, VG, USA); after reaching approximately 80% confluence, culture medium was decanted from the plates and the cells were intoxicated with solutions of test compounds. Concentrations of 25 μM each of quercetin, curcumin, and CAPE were used. After 24 h of culture, staining was started. First, viable cells were washed in PBS (3 × 5 min, 37 °C) and then incubated in LysoTracker Red DND-99 (Molecular Probes, Eugene, OR, USA, L 7528) prepared in PBS for 20 min at 37 °C under dark conditions. After staining, the culture was washed again in PBS (3 × 5 min) and stained with Hoechst 33342 reagent (Life Technologies) for 15 min at 37 °C in the dark. The slides were resealed with VECTASHIELD (Vector Laboratories) mounting medium the analysis was started with the OLYMPUS BX60 microscope using the appropriate filters. Photographic documentation was made using the XC50 digital camera (Olympus) and cellSens Standard computer software (Olympus, ver. 1.8.1). The quantitative evaluation of fluorescence was performed using ImageJ Software version 1.53 m (NIH, Bethesda, MD, USA). Each sample image was processed in a lookup table (LUT), with lookup of the red or green hot spots, respectively. Higher mean values indicated higher intensity of the wave response.

### 4.6. Detection of Apoptotic Cells by the TUNEL Method

The TUNEL method (Terminal Deoxynucleotidyl Transferase Mediated d-UTP Nick End-Labeling), which allows the detection of apoptotic cells, is based on the detection of DNA fragmentation. An important feature of cells undergoing apoptosis is the fragmentation of DNA into segments equal to multiples of the length of nucleosomes. Differences in the method of DNA degradation are the basis for differentiating between necrotic and apoptotic death. In the case of necrotic fragmentation, random degradation of the genetic material occurs, forming a characteristic smudge on the agarose gel. However, in the case of apoptosis DNA fragmentation is observed instead, reflecting the structure of histone octamers, as a result of which the characteristic ladder of DNA fragments appears on the agarose gel. During apoptosis, many single- or double-stranded breaks are generated as a result of DNA digestion. Their marking is the basis of the TUNEL method. Terminal transferase (TdT) adds digoxigenin-labelled nucleotides to the free hydroxide ends without the presence of a template, resulting in coloured products [126,127].

To detect apoptotic cells using the TUNEL method, cells were plated in six-well plates (Corning™, Mediatech, Inc., Manassas, VG, USA). After obtaining the appropriate confluence of the culture, the cells were treated with quercetin, curcumin, and CAPE each at concentrations of 25 μM. Cultivation was continued for 24 h. The cells were then fixed in 4% paraformaldehyde (40 min, 37 °C) and washed with TBS (TRIS buffered saline, pH 7.6) three times for 5 min. After washing, the cells were incubated with 0.1% Triton X-100 (Sigma, Kawasaki City, Japan) in TBS for 5 min, then 0.1% Triton X-100 in 0.1% citrate buffer was added to the culture. After 2 min incubation at 4 °C, the cells were washed with TBS solution (3 × 5 min) and stained in the dark with the TUNEL reaction mixture (d-UTP with terminal deoxynucleotide transferase) (In Situ Cell Death Detection Kit, TMR red, Roche, Basel, Switzerland) for 1 h at room temperature. After staining, the cell monolayer was washed again in TBS solution (3 × 5 min) and stained with DAPI (4,6-diamidino-2-phenylindole) reagent at room temperature for 15 min under dark conditions. In accordance with the In Situ Cell Death Detection Kit (Roche) protocol, a negative test was performed in this experiment as well. Stained slides were sealed with VECTASHIELD mounting medium (Vector Laboratories) and analyzed with an OLYMPUS BX60 microscope using appropriate filters. Micrographs were taken using an XC50 digital camera (Olympus) and cellSens Standard computer software (Olympus, ver. 1.8.1). As a result of the reaction, stained red signals within the nuclei of apoptotic cells and blue DNA fluorescence were observed. The quantitative evaluation of fluorescence was performed using ImageJ Software version 1.53 m (NIH, Bethesda, MD, USA). Each sample image was processed via lookup table (LUT), with the red or green hot spots used for lookup, respectively. Higher mean values indicate higher intensity of the wave response.

### 4.7. Detection of Reactive Oxygen Species

In order to detect reactive oxygen species (ROS), a kit with dihydroethidium (DHE) was used. Due to its ability to freely cross cell membranes DHE is often used to monitor O_2_^•−^ production, and can be used to study respiratory bursts in cells. DHE is considered to be the most specific dye for ROS detection because it is well tolerated by cells and does not break down after the mild fixation used during microscopic preparation [128].

ROS in the test line was detected by subculture of the cells into six-well plates (Corning™, Mediatech, Inc., Manassas, VG, USA), and after reaching appropriate confluence, intoxication with the tested flavonoids. The following concentrations were used in the study: 25 μM each of quercetin, curcumin, and CAPE. After 24 h of cultivation, the experiment was started. Viable cells were washed with PBS (3 × 5 min, 37 °C) and incubated with DHE dye (Invitrogen) prepared in PBS buffer for 20 min at 37 °C in the dark. After incubation, the cells were washed again in PBS (3 × 5 min) and stained with Hoechst 33342 (Life Technologies, Carlsbad, CA, USA) reagent (15 min, 37 °C, in the dark). The preparations prepared in this way were sealed with VECTASHIELD (Vector Laboratories, USA) mounting medium and analysed in the OLYMPUS BX60 microscope using appropriate filters. Micrographs were taken using an XC50 digital camera (Olympus). The image was developed in cellSens Standard computer software (Olympus, ver. 1.8.1). The quantitative evaluation of fluorescence was performed using ImageJ Software version 1.53 m (NIH, Bethesda, MD, USA). Each sample image was processed via lookup table (LUT), with the red or green hot spots used for lookup, respectively. Higher mean values indicate higher intensity of the wave response.

### 4.8. Electron Microscopy

LNCaP cells treated with the tested compounds were observed by electron microscopy to visualize ultrastructural changes. Cells grown in six-well plates (CorningTM, Mediatech, Inc., Manassas, VG, USA) and intoxicated with selected concentrations of quercetin, curcumin, and CAPE (25 μM each) were subjected to routine preparation, allowing for observation via transmission electron microscope.

The cells were fixed in a 2.5% paraformaldehyde solution prepared in phosphate buffer. Fixation was carried out for 72 h. The prefixed material was washed with 0.1 M phosphate buffer pH 7.4 for 30 min. Subsequently, a 1% osmium tetroxide solution (Polyscience Inc., Warrington, PA, USA) in phosphate buffer was refixed for 20 min in the dark. The OsO4-fixed material was rinsed twice with phosphate buffer, then dehydrated and impregnated with epoxy resin. A series of ethanol solutions with increasing concentration gradients and acetone were used for dehydration. The infiltration of the cells with a 1:1 mixture of epon and acetone was carried out for 2 h at room temperature. After this time, the culture plates were opened to let the acetone evaporate. The next day, the embedding medium was changed to an acetone-free eponic mixture. After embedding the cells, the epon mixture polymerized for 72 h at 60 °C. Poly/Bed (R) 812 Embeeding Media/DMP-30 Kit epoxy resin (Polyscience, Inc., Warrington, PA, USA) was used in the tests. Epon blocks with embedded cellular material were trimmed and sectioned using a Leica Ultracut UCT ultramicrotome. Semi-thin sections were collected (approx. 0.5 μm thick) on glass slides and hot-stained with 1% methylene blue solution to localize the cell monolayer. The blocks were then prepared with a diamond knife (DiATOME^®^ Ultra Diamond Knife, Diatome USA, Hatfield, PA, USA) to obtain ultrathin sections of 50–70 nm. Ultrathin sections were placed on 300 Mesh copper grids (Agar Grids 300 Mesh Copper 3.05 mm; Agar Scientific Ltd., Stansted, UK). Slides on the meshes were contrasted with a 13% uranyl acetate solution for 5 min in the dark, then rinsed several times in 50% ethyl alcohol. The next stage of the experiment consisted of staining the sections with a solution of lead citrate for 10 min in a chamber with a sodium hydroxide crystal. Contrasting was completed by rinsing several times in distilled water and drying the slides at room temperature. The material prepared in this way was analyzed using a Hitachi H500 transmission electron microscope at an accelerating voltage of 75 kV. The documentation was made on KODAK photographic plates.

### 4.9. Statistical Analysis

All results are presented as mean ± SD calculated from three independent experiments and performed in quadruplicate (*n* = 12). Results were obtained using independent samples *t*-tests. Experimental mean values (each individually) were compared to mean values of untreated cells (control) harvested in parallel for 24 and 48 h, respectively. In addition, we compared the difference between 24 h and 48 h incubation times at the same concentration. Differences were tested for significance using one-level or multilevel Levene and Friedman analysis of variance tests.

## 5. Conclusions

The results of this research allow us to formulate the following conclusions:

1. The tested compounds obtained from bee products have antiproliferative and cytotoxic activity against LNCaP prostate cancer cells.
2. The tested compounds show partially dose- and time-dependent effects on mitochondrial and lysosomal activity as well as on total protein synthesis in the test cells.
3. Quercetin, the strongest of the tested compounds, reduces the mitochondrial activity of LNCaP cells and inhibits the synthesis of cell proteins.
4. Among the tested compounds, curcumin shows the strongest effect on the lysosomal activity of prostate cancer cells.
5. Preliminary studies suggest that the cytotoxic effect of quercetin is based on the induction of apoptosis in LNCaP cells, while CAPE induces autophagy processes in cells and curcumin causes morphological changes suggesting necrosis of prostate cancer cells.
6. Due to their biological activity, selected flavonoids could be used in the prevention and chemotherapy of cancer; however, this requires further research.

## 6. Patents

No patents related to the results of these studies are pending.

## Figures and Tables

**Table 1:** IC50 values of the tested compounds for the XTT-SRB-NR test at 24 and 48 h. * statistically significant decrease in viability relative to control (*p* < 0.05).

Compound	Time	XTT Test (μM)	NR Test (μM)	SRB Test (μM)
CAPE	24 h	90.15 *	200.60 *	59.93 *
	48 h	179.81 *	277.79 *	24.93 *
Curcumin	24 h	121.99 *	96.86 *	89.24 *
	48 h	48.80 *	90.71 *	71.69 *
Quercetin	24 h	78.43 *	133.65 *	<10.00 *
	48 h	<10.00 *	201.20 *	40.98 *

## Data Availability

All data supporting the reported results can be obtained on request from the corresponding author.
